# Genetic Algorithm for Feature Selection in Lower Limb Pattern Recognition

**DOI:** 10.3389/frobt.2021.710806

**Published:** 2021-10-25

**Authors:** Robert V. Schulte, Erik C. Prinsen, Hermie J. Hermens, Jaap H. Buurke

**Affiliations:** ^1^ Roessingh Research and Development, Enschede, Netherlands; ^2^ Department of Biomedical Signals and Systems, University of Twente, Enschede, Netherlands; ^3^ Department of Biomechanical Engineering, University of Twente, Enschede, Netherlands

**Keywords:** genetic algorithm, feature selection, pattern recognition, myoelectric control, lower limb, intent recognition

## Abstract

Choosing the right features is important to optimize lower limb pattern recognition, such as in prosthetic control. EMG signals are noisy in nature, which makes it more challenging to extract useful information. Many features are used in the literature, which raises the question which features are most suited for use in lower limb myoelectric control. Therefore, it is important to find combinations of best performing features. One way to achieve this is by using a genetic algorithm, a meta-heuristic capable of searching vast feature spaces. The goal of this research is to demonstrate the capabilities of a genetic algorithm and come up with a feature set that has a better performance than the state-of-the-art feature set. In this study, we collected a dataset containing ten able-bodied subjects who performed various gait-related activities while measuring EMG and kinematics. The genetic algorithm selected features based on the performance on the training partition of this dataset. The selected feature sets were evaluated on the remaining test set and on the online benchmark dataset ENABL3S, against a state-of-the-art feature set. The results show that a feature set based on the selected features of a genetic algorithm outperforms the state-of-the-art set. The overall error decreased up to 0.54% and the transitional error by 2.44%, which represent a relative decrease in overall errors up to 11.6% and transitional errors up to 14.1%, although these results were not significant. This study showed that a genetic algorithm is capable of searching a large feature space and that systematic feature selection shows promising results for lower limb myoelectric control.

## 1 Introduction

Motor intent recognition using electromyography (EMG) has the potential to create intuitive control of prosthetic devices. However, EMG signals are noisy in nature, which makes it challenging to extract user intent (Phinyomark and Scheme, 2018). To solve this challenge, feature extraction can be used to improve information density and reduce noise, which leads to better intent recognition. Numerous feature extraction methods and feature combinations have been proposed (Phinyomark and Scheme, 2018; Hudgins et al., 1993; Phinyomark et al., 2017; Phinyomark et al., 2018). The best combination of features can be found by trying out every combination; but with the increase in possible features over the years, this becomes unfeasible. Therefore, feature selection and dimension reduction techniques have been used to remove redundant and irrelevant features.

However, big datasets require more complex methods as classical methods are not sufficient due to their computational complexity or lack of the ability to circumvent sub-optimal solutions (Phinyomark and Scheme, 2018). Therefore, feature selection methods have been proposed, which are capable of searching large-dimensional spaces (Phinyomark and Scheme, 2018). Meta-heuristics, such as the genetic algorithm (GA) (Luo et al., 2017; Karthick et al., 2018; Xi et al., 2018; Rong et al., 2013; Too et al., 2019), were used for feature selection or optimization in EMG-based classification. A genetic algorithm is known for its efficient search of large, possibly noisy solution spaces (Huang and Wang, 2006; Krömer et al., 2018), and due to its simplicity and ease of interpretation, it is used in many applications (Krömer et al., 2018). Luo et al. (2017) applied a genetic algorithm for feature selection in movement classification based on EMG. They used a multi-layer perceptron and 10 initial features per channel. The authors showed that the genetic algorithm is capable of reducing the number of necessary features while reaching a higher classification accuracy and improving state-of-the-art by increasing the accuracy of hand gesture recognition from 91 to 97.7%. Xi et al. (2018) investigated motor intent recognition using EMG in the lower limb, focusing on eight different activities of daily living. They compared feature sets selected with different methods, ranging from plain correlation analysis to genetic algorithm–weighted correlation analysis. Using the genetic algorithm, they were able to reduce the number of features, increase the quality of the feature set, and increase the classification accuracy in combination with a support vector machine. Compared with correlation analysis, they improved the specificity from 69.5–72.5 to 95.9–100% and sensitivity from 67.2–70.6 to 96.6–100%. Rong et al. (2013) investigated muscle fatigue classification, comparing neural networks, support vector machines, and an optimized support vector machine using a genetic algorithm. They concluded that a genetic algorithm can optimize support vector machine parameters to increase classification accuracy, from 66.1% up to 97.6%. Halim et al. (2020) used a genetic algorithm to reduce the number of sensors necessary to reach similar performance versus all sensors in the lower limb. They showed that, with only a decrease of 0.9% in accuracy from 94.0 to 93.1%, they could reduce the number of sensors necessary by 54%. Other meta-heuristics were used in EMG feature selection as well (Phinyomark and Scheme, 2018), such as particle swarm optimization (Purushothaman and Vikas, 2018; Too et al., 2019; Zhang et al., 2019; Bakiya et al., 2020) and ant colony optimization (Purushothaman and Vikas, 2018). Many other meta-heuristics exist which are suited for feature selection in general, such as tabu search and simulated annealing (Diao and Shen, 2015). These studies show that meta-heuristics and especially genetic algorithms are capable of efficiently improving classification performance.

The number of features used in each study, at most fifteen, is limited compared with the hundreds of possible feature extraction methods that have been described in the literature. Phinyomark et al. (2017) identified 58 different feature extraction methods (Phinyomark et al., 2017; Côté-Allard et al., 2020), which range from time- to frequency-domain features. In their work, they evaluated individual features to see in which “category” the features belong using a mapping method and, in this way, constructed a feature set based on their topology. They showed that their method outperforms sequential feature selection. The downside of this method is that information of the observer is necessary to evaluate the groups and construct a feature set that is based on these groups. A meta-heuristic, such as the genetic algorithm, would make the search more objective and could perform feature selection on its own. However, the question arises whether an optimal solution could be found when the number of features is high, as many possible combinations exist within the solution space. Furthermore, most of the work on feature selection in myoelectric control is performed on the upper limb. Various studies adapt the optimized feature sets for the upper limb to be used in the lower limb, but the question arises whether the use of upper limb features would suffice for control within the lower limb.

In this work, we implemented a genetic algorithm to search a large feature space to design a feature set suited for myoelectric control in the lower limb. The expectation is that the feature set designed by the genetic algorithm outperforms current state-of-the-art feature sets.

## 2 Materials and Methods

### 2.1 Data

Two datasets were used within this study. The first is the ENABL3S dataset containing 10 able-bodied subjects (7 m, 3 f) which was collected by Hu et al. (2018a). This dataset contains EMG, joint angle, acceleration, and angular velocity data. EMG was collected using bipolar electrodes, from seven muscles per leg: rectus femoris, vastus lateralis, biceps femoris, semitendinosus, tibialis anterior, gastrocnemius medialis, and soleus. Joint angles in the sagittal plane were collected using electrogoniometers of the knee and ankle, and 3D acceleration and 3D angular velocity were measured using IMUs on the lower and upper legs. Data were collected of sitting, standing, walking, stair ascent/descent, and ramp ascent/descent.

**FIGURE 1:**
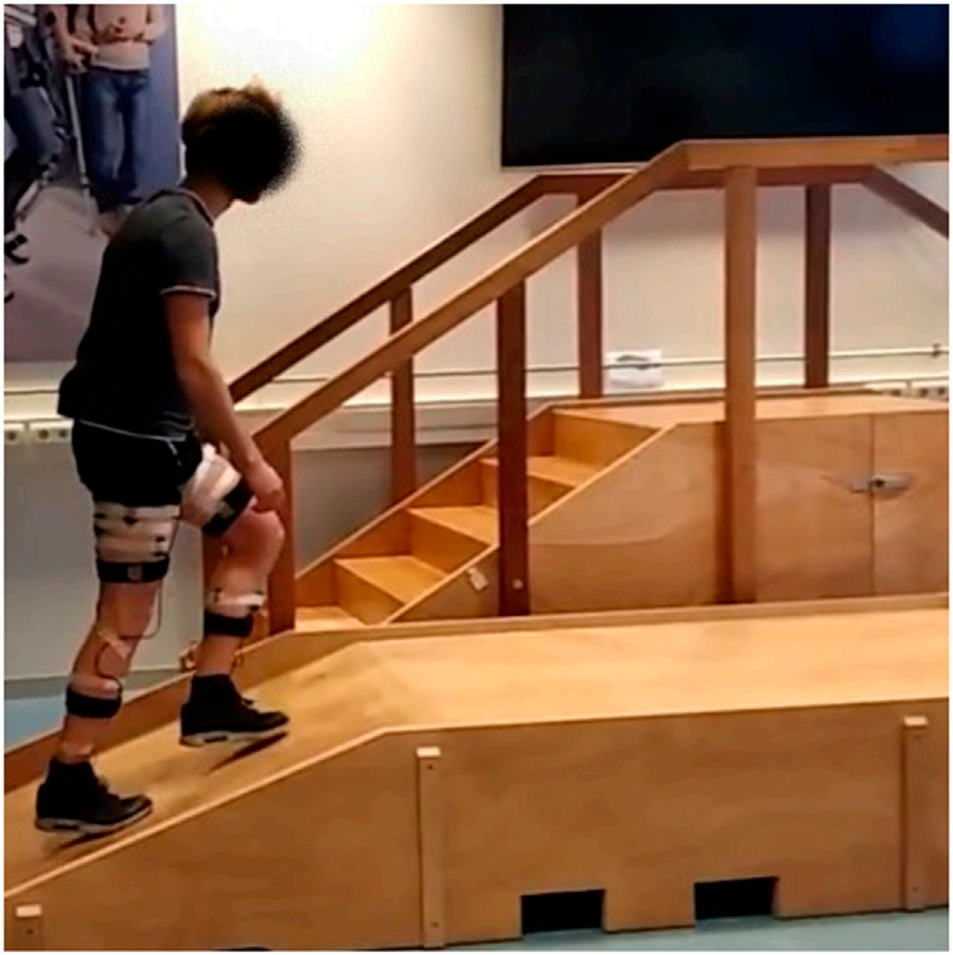
Measurement setup of the MyPredict dataset, with the ramps and stairs.

The second is the MyLeg-Roessingh database for activity prediction (MyPredict) containing 10 able-bodied subjects (7 m, 3 f), which was collected for this study. The protocol was reviewed and approved by Medical research Ethics Committees United (MEC-U) Nieuwegein, Netherlands. The participants provided their written informed consent to participate in this study. This dataset contains EMG, joint angle, acceleration, and angular velocity data. EMG was collected using bipolar electrodes (Delsys, Boston, United States), from eight muscles per leg: gluteus maximus, gluteus medius, rectus femoris, vastus lateralis, biceps femoris, semitendinosus, tibialis anterior, and gastrocnemius medialis. Signals were recorded at 1000 Hz. Lower body kinematics were collected using an MVN Link suit (Xsens, Enschede, Netherlands), which uses eight inertial measurement units (IMUs) to reconstruct lower body movement at 240 Hz. IMUs were placed on the feet, lower legs, upper legs, pelvis, and sternum. In this work, we used the 3D joint angles from the hip, knee, and ankle and the 3D acceleration and 3D angular velocity of the feet, lower legs, and upper legs. All data were time synchronized and resampled to 1000 Hz. EMG was filtered with a zero-lag second-order Butterworth high-pass filter with a cut-off frequency of 20 Hz. Measurements were conducted at the Wearable Robotics Lab of the University of Twente, using obstacles constructed for the Cybathlon by the Department of Biomechanical Engineering, see Figure 1. Before each measurement, the maximal voluntary contraction of each muscle was measured to normalize EMG. Obstacles used were the stairs (rise 17 cm, run 28 cm), ramp with two different slopes (15 and 20°), and uneven terrain consisting of stepping stones on a surface. Forty trials were conducted per subject. A trial consisted of sitting, standing, walking, stair ascent, walking, stair descent, walking, ramp ascent, walking, ramp descent, walking, walking on uneven terrain, walking in confined spaces, walking, standing, and sitting. Subjects walked at their own preferred speed, and after ten trials, a small break was administered to avoid fatigue and check sensor placement. Each trial had a duration of around 1 min 15 s. The total measurement time including subject preparation, sensor placement, and calibration was around 2 h.

We used data of gait-related activities which were present in both datasets to have comparable data in both datasets. These activities were standing, walking, stair ascent/descent, and ramp ascent/descent.

### 2.2 Genetic Algorithm

Genetic algorithms search the solution space using a nature-inspired process based on genetic evolution. The genetic algorithm uses a population of candidate solutions, which are evaluated based on their fitness. Solutions with a higher fitness are more likely to continue in the genetic process, whereas poor solutions are phased out. These solutions encode the problem at hand and are comparable with chromosomes containing certain genes. These “chromosomes” undergo crossover and mutation operations to evolve into a new population of solutions. This process is repeated until the process has converged to a solution or the maximum number of iterations is reached.

#### 2.2.1 Problem Encoding

An important step in genetic algorithm design is problem encoding. In our case, the solutions were feature sets leading to a certain accuracy. We encoded features into a binary string. If the solution contained a certain feature, the value for that feature will be 1. If not, then the value will be 0. In this way, we encode every feature for every data type. The datasets contained four different data types: joint angles (ang), acceleration (acc), angular velocity (gyr), and EMG (emg). Therefore, the chromosome looked as follows:

$$chromosome=\left(\begin{array}{c} F_{ang}\\ F_{acc}\\ F_{gyr}\\ F_{emg} \end{array}\right)=\left(\begin{array}{cccc} f_{1} & f_{2} & \ldots & f_{N}\\ f_{1} & f_{2} & \ldots & f_{N}\\ f_{1} & f_{2} & \ldots & f_{N}\\ f_{1} & f_{2} & \ldots & f_{N} \end{array}\right)\text{ with }f_{n}\in \left\{0,1\right\}.$$ (1)

Here, *F* is the feature set for each data type and *f* is the feature extraction method.

#### 2.2.2 Fitness

Another important part of the genetic algorithm is the fitness function. The fitness function determines the fitness of the solution, which leads to the probability of the solution to continue in the evolutionary process. The fitness function used in this work is specific to this application. For lower limb myoelectric pattern recognition, three performance metrics are important, which are the overall performance, the steady-state performance, and the transitional performance. A fitness function should reflect these important factors for lower limb pattern recognition. Next for performance, it is important that a feature set would not be too large as using this feature set would take up too much time to train and classify. Therefore, we implemented the following fitness function:

$$fitness=\alpha_{ov}{\bar{s}}_{ov}+\alpha_{ss}{\bar{s}}_{ss}+\alpha_{tr}{\bar{s}}_{tr}+\frac{\beta }{n_{f}}.$$ (2)

Here, $\bar{s}$ is the average score over all subjects for overall (*ov*), steady-state (*ss*), and transitional (*tr*) performances. The performance was determined using the classification accuracy of the mode-specific linear discriminant analysis (LDA) classifiers, as described by Hu et al. (2018b). *α* is a scaling factor that determined the importance of each performance type and was set to 0.25, 0.1, and 0.5 in this work for *ov*, *ss*, and *tr*, respectively. The data contain mostly steady states, which means that the overall performance is mostly determined by the steady-state performance and not by the transitional performance. To balance this out, the transitional performance was given a higher weight to counteract the influence of steady states, as steady states occur more in the data. If the values for transitions were too high, the overall error would rise, which is not preferable as well. *n*_*f*_ is the number of features used for the chromosome, and *β* is a scale factor. $\frac{\beta }{n_{f}}$ determined the fitness based on the number of features used: the lower the number of features, the higher the fitness value with a minimum number of features of 1. This part of the fitness function is implemented to steer the genetic algorithm away from very large feature sets. *β* was set to 0.15 in this work as it should not be considered the most prominent selection criterion, as performance is considered more important. With these scale factors, the maximal possible fitness is 1.00.

#### 2.2.3 Crossover

Crossover determines how two selected chromosomes are combined to form a new chromosome for the next generation. These parent chromosomes are selected based on their fitness value, using roulette wheel selection. When the parents are chosen, two children are created from the pair. For each data type, a crossover point is randomly selected and the first part of the first child is coming from parent 1 and the second part from parent 2. The remaining parts of the parents form the second child. The process of randomly combining parents was repeated until the new population was formed. The population size was set to 64, and the number of parents per iteration was set to 16.

#### 2.2.4 Mutation

Mutation alters a new chromosome by point mutation, e.g., changing a 0 to a 1 or vice versa. The parameter mutation rate determines if and how often a chromosome was mutated. In this work, we implemented a dynamic mutation rate: the mutation rate started out at 10%, and if the best fitness value did not change for 10 iterations, we increased the mutation rate by 5% up to 20%. If the fitness increased during 10 iterations, the mutation rate was decreased by 5% up to as minimum 10%. The search was stopped if the best fitness value did not increase after another 10 iterations when the highest mutation rate was reached. Next to that, the other stopping criterion was the maximum number of iterations, which was set to 200.

#### 2.2.5 Heterogeneity

From initial tests, it seemed that the population converged quickly to a homogeneous population in terms of fitness. Therefore, we implemented a check based on the interquartile range of the fitness of the population: if the interquartile range was below 0.002, we would add eight random chromosomes to the population. To ensure a similar size of the population during iterations, eight fewer children were created during crossover.

### 2.3 Feature Extraction and Evaluation

When using feature selection or hyperparameter optimization, one of the major risks is overfitting, and therefore, it is important to implement a proper evaluation strategy (Halilaj et al., 2018). To demonstrate the generalizability of the approach, we optimized the feature set on 80% of the MyPredict dataset which was collected for this study and tested this feature set on the remaining 20% of the MyPredict dataset. By using the last 20%, we try to mimic a real-life setting, i.e., data that were collected after the training phase. Next to this, performance was also evaluated on a completely separate dataset, the ENABL3S dataset. During optimization, a fourfold cross validation was used to determine the fitness of each chromosome per subject, as this provides a good trade-off between speed and accuracy. For final evaluation on the ENABL3S dataset, a 10-fold cross validation was applied to determine the performance of the feature set, similarly to that done by Hu et al. (2018b).

The feature extraction methods are described in Table 1. In total, 62 feature extraction methods were implemented, resulting in 67 features. Certain features contain more than one value, such as autoregressive coefficients (ARCs). The individual values of such a feature are not taken into account during the feature selection process. That is, all values of ARCs were used if ARCs were selected.

**TABLE 1:** The 62 feature extraction methods used in this work. This table is based on the work of Phinyomark et al. (2017), Hu et al. (2018b), and Atzori et al. (2014).

Name	Abbreviation	Notes	Size
Amplitude of the first burst	AFB	wf = 32 ms	1
Approximate entropy	ApEn	—	1
Autoregressive coefficients	ARC	order = 4	5
Cepstrum coefficients	CC	order = 4	5
Critical exponent analysis	CEA	—	1
Differenced version of ARC	DARC	order = 4	5
Differenced version of CC	DCC	order = 4	5
Detrended fluctuation analysis	DFA	—	1
Differenced version of LOG	DLD	—	1
Differenced version of MAV	DMAV	—	1
Max-to-min drop in PSD ratio	DPR	—	1
Differenced version of STD	DStd	—	1
Differenced version of TM	DTM	order = 3	1
Differenced version of V	DV	order = 3	1
Differenced version of VAR	DVAR	—	1
End value	EndVal	—	1
Frequency ratio	FR	f_*lb*_ = [20−45], f_*hb*_ = [95−f_*max*_]	1
Higuchi fractal dimension	HG	kmax = 128	1
Histogram	HIST	bins = 3, 10	3, 10
Integrated EMG	IEMG	—	1
Katz fractal dimension	KATZ	—	1
Kurtosis	KURT	—	1
Log detector	LD	—	1
Second-order moment	M2	—	1
Mean absolute value	MAV	—	1
MAV type 1	MAV1	—	1
MAV type 2	MAV2	—	1
Maximal value	MAX	—	1
Marginal discrete wavelet transform	mDWT	wavelet = db7, level = 3	3
Mean	MEAN	—	1
Minimal value	MIN	—	1
Mean frequency	MNF	—	1
Mean power	MP	—	1
Myopulse percentage rate	MYOP	Threshold 2e1/2e-2/5e-5	1, 1, 1
Power spectrum deformation	OHM	—	1
Peak frequency	PKF	—	1
Power spectrum fractal dimension	PSDFD	using katz	1
Power spectrum ratio	PSR	—	1
Root mean square	RMS	—	1
Sample entropy	SampEn	—	1
Skewness	SKEW	—	1
Spectral moment	SM	order = 2	1
Signal-to-motion ratio	SMR	frequency: <10 Hz	1
Signal-to-noise ratio	SNR	—	1
Number of slope sign changes	SSC	—	1
Simple square integral	SSI	—	1
Start value	StartVal	—	1
Standard deviation	STD	—	1
Time-dependent power spectrum descriptors	TDPSD1-6		1,1,1,1,1,1
Absolute temporal moment	TM	order = 3	1
Total power	TP	—	1
v-order	V	order = 3	1
Variance	VAR	—	1
Variance of central frequency	VCF	—	1
Willison amplitude	WAMP	Threshold 2e1/2e-2/5e-5	1, 1, 1
Waveform length	WL	—	1
Number of zero crossings	ZC	—	1

The genetic algorithm ran ten times on all sensor modalities, each time potentially selecting a different optimal feature set due to its stochastic nature. The best performing feature set (GA-Opt) was considered for final evaluation on the test sets. Next to GA-Opt, other feature sets were constructed as well. These feature sets were based on the occurrence of the features. For example, if a feature was selected in at least six out of ten runs, it was added to the feature set GA-06. If a feature was selected at least twice, it was added to the feature set GA-02. Out of these feature sets, one feature set was selected for final evaluation on the test sets based on its performance on 80% of the MyPredict dataset.

The genetic algorithm ran per modality as well, to select features per modality instead of selecting features of all modalities at once. The genetic algorithm ran 10 times per sensor modality (angle, acceleration, angular velocity, EMG). The best performing feature sets of each modality were combined into a new feature set (GM-Opt). As described before, feature sets were created based on occurrence as well. The best performing feature set based on occurrence was selected for the final evaluation on the test sets.

Finally, the four genetic optimized feature sets were compared against the best performing state-of-the-art feature set. The best performing state-of-the-art feature set was determined by the performance of the feature set on 80% of the MyPredict dataset. Six state-of-the-art feature sets were considered, described by Atzori et al. (2014), Hu et al. (2018b), Hudgins et al. (1993), Phinyomark et al. (2013), Phinyomark et al. (2017) and Khushaba et al. (2014), which are shown in Table 2. The best performing feature set was selected for final evaluation on the test sets.

**TABLE 2:** Six state-of-the-art feature sets used in myoelectric control. In this case, the feature set used by Hu et al. (2018b) was the only set that contained separate features for kinematic data. Feature definitions can be found in Table 1.

Name	Features
Atzori et al. (2014)	mDWT, HIST10, RMS, MAV, WL, SSC, ZC
Hu et al. (2018b)	EMG: MAV, WL, SSC, ZC, ARC kinematics: MEAN, STD, MIN, MAX, StartVal, EndVal
Hudgins et al. (1993)	MAV, WL, SSC, ZC
Phinyomark et al. (2013)	SampEn, CC, RMS, WL
Phinyomark et al. (2017)	DMAV, DStd, WAMP1, ZC, SampEn, MFL, DARC, TDPSD1-6
Khushaba et al. (2014)	TDPSD1-6

The implemented classifiers were subject-specific, mode-specific LDA classifiers as described by Hu et al. (2018b). The features were extracted from windows of 300 ms before each gait event. Hereafter, the features were scaled to have zero mean and unit variance. Principal component analysis retaining 95% of the variance was used to reduce dimensions before classifying the data with mode-specific LDA classifiers, as done by Hu et al. (2018b). We only looked at the performance in terms of an ipsilateral sensor setup, to mimic use within a prosthetic device. Only kinematic sensors on the legs and feet were used, and thus, pelvis/sternum kinematics were excluded. Performance was split into overall, steady-state, and transitional errors. A step was considered a transition when the state of the previous gait event differed from that of the current gait event. As most of the data were from steady states, the overall error was primarily influenced by steady-state errors, and therefore, transitional errors are shown separately. Feature extraction methods and the genetic algorithm were implemented in Python 3.9. Code can be found at https://github.com/Rvs94/GeneticAlgorithmForFS.

### 2.4 Statistic Analysis

Feature set error rates were compared with a repeated-measures ANOVA. The pairwise t-test with Sidak correction was used to determine significant differences between feature sets. Normality was visually inspected and confirmed using a Shapiro–Wilk test. All analyses were performed within IBM SPSS version 27.

## 3 Results

### 3.1 Selected Features—All Modalities

**FIGURE 2:**
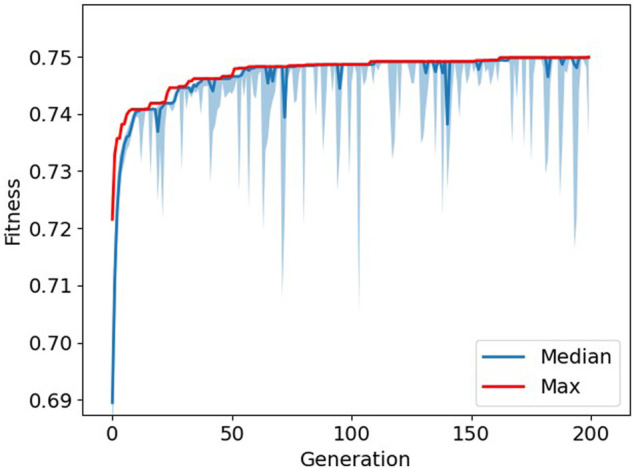
Progression of fitness during optimization. The maximum fitness is depicted in red, and the median fitness with interquartile ranges is depicted in blue. The larger “spikes” in the interquartile range show where the GA introduced new random samples to the population.

The average fitness over ten runs of the genetic algorithm was 0.749 ± 0.001. The number of iterations differed per run, ranging from 143 to 200 iterations. The average number of features selected was 14.1 ± 2.2, 10.4 ± 1.7, 8.6 ± 2.3, and 5.7 ± 2.0 for joint angle, acceleration, angular velocity, and EMG data, respectively. An example of the optimization process can be seen in Figure 2. Out of these ten runs, the feature set with the highest fitness value was considered to be GA-Opt. GA-Opt had a fitness value of 0.750, containing 16, 11, 11, and 6 features for joint angle, acceleration, angular velocity, and EMG data, respectively, see also Table 3. The average error rates and standard error of the mean (SEM) were 4.57 ± 0.25% and 16.47 ± 1.22% for overall and transitional errors, respectively, on 80% of the MyPredict dataset.

**TABLE 3:** Selected features of GA-Opt and GA-03 for each data type. The occurrence of the features is given after each feature for GA-03. Feature definitions can be found in Table 1.

Type	GA-Opt	GA-03 (occurrence)
ang	AFB, DFA, DLD, DV, DVAR, EndVal, IEMG, M2, MAV, MAV2, OHM, SSI, STD, TP, VAR, WAMP2	EndVal (8), WL (6), mDWT (6), LOG (5), TP (5), VAR (5), CEA (4), DLD (4), DV (4), MAX (4), RMS (4), SSI (4), V (4), VCF (4), AFB (3), DMAV (3), DVAR (3), M2 (3), MAV (3), MP (3), SampEn (3), StartVal (3)
acc	AFB, DMAV, IEMG, LOG, MAV, MIN, MYOP3, OHM, SKEW, SSC, SampEn	LOG (6), SSI (6), MEAN (5), AFB (4), DStd (4), IEMG (4), MAV (4), MYOP3 (4), OHM (4), WL (4), DV (3), HIST3 (3), RMS (3), SKEW (3), SampEn (3), TM (3), VAR (3)
gyr	AFB, CEA, DFA, DMAV, M2, MEAN, MNF, SMR, SSI, VAR, WAMP3	Mean (7), MAX (6), MYOP3 (5), DStd (3), EndVal (3), M2 (3), MAV (3), MYOP2 (3), TM (3), VAR (3)
emg	CEA, DStd, SKEW, SSC, STD, ZC	WAMP2 (5), ZC (4), DStd (3), KATZ (3), PSDFD (3)

**FIGURE 3:**
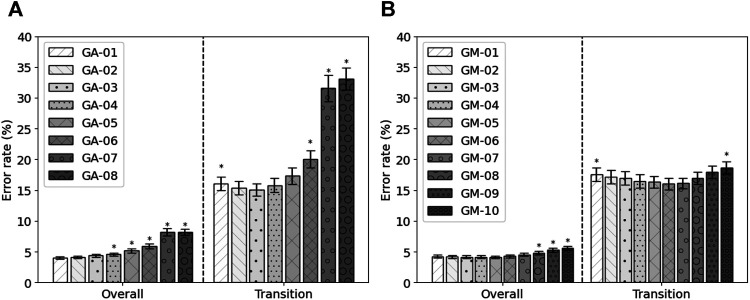
Average error rates (± SEM) of the constructed GA feature sets **(A)** and of the constructed GM feature sets **(B)** on 80% of the MyPredict dataset. A star indicates a significant difference compared with the best performing feature set. For the GA feature sets **(A)**, the best performing feature set was GA-01 for overall error, and in case of transitional errors, this was GA-03. For the GM feature sets **(B)**, the best performing feature set overall was GM-05, and for transitional errors, this was GM-06.

Next to the feature set with the highest fitness, other feature sets were constructed. Based on the occurrence of the features within the runs of the genetic algorithm, eight feature sets were constructed, GA-01 to GA-08. No feature occurred more than eight times. GA-01 contained all features that were selected at least once, GA-02 contained all features that were selected at least twice, etc. No feature was selected more than eight times. Their performance is shown in Figure 3A. It can be seen that, in terms of overall error, GA-01 performs best. No significant differences were found with GA-02 and GA-03. GA-04 up to GA-08 perform significantly worse than GA-01 (*p* = 0.002, *p* = 0.002, *p* = 0.0009, *p* < 0.0003, p < 1e−4, respectively). For transitional errors, GA-03 performs best. No significant differences were found with GA-02, GA-04, and GA-05. GA-01 (*p* = 0.01), GA-06 (*p* = 0.008), GA-07 (*p* = 0.0001), and GA-08 (p < 1e−4) perform significantly worse than GA-03. As GA-03 is not significantly worse than GA-01 in terms of overall error while having a smaller number of features and performs best in terms of transitional errors, GA-03 was considered to be the best performing feature set out of these eight constructed feature sets. The average error rates of GA-03 were 4.37 ± 0.25% and 15.06 ± 1.01% for overall and transitional errors, respectively, on 80% of the MyPredict dataset.

### 3.2 Selected Features—Per Modality

The average fitness value per modality was 0.738 ± 0.003 for angles, 0.731 ± 0.002 for acceleration, 0.703 ± 0.003 for angular velocity, and 0.647 ± 0.014 for EMG data. The number of iterations was 106–200 for angles, 84–200 for acceleration, 139–200 for angular velocity, and 109–200 for EMG data. Per modality, the run with the highest fitness value was considered the optimal feature set for that modality. Hereafter, these feature sets were combined to form GM-Opt. GM-Opt contained 9, 25, 25, and 5 features for angles, acceleration, angular velocity, and EMG, respectively. The features of GM-Opt are shown in Table 4.

**TABLE 4:** Selected features of GM-Opt and GM-06 for each data type. The occurrence of the features is given after each feature for GM-06. Feature definitions can be found in Table 1.

Type	GM-Opt	GM-06 (occurrence)
ang	DV, DVAR, EndVal, RMS, SM, SMR, StartVal, VAR, mDWT	mDWT (10), DVAR (7), StartVal (7), DV (6), EndVal (6)
acc	AFB, CEA, DMAV, EndVal, HIST10, LOG, MAV1, MEAN, MIN, MP, MYOP2, OHM, RMS, SNR, SSC, SSI, STD, TDPSD1, TDPSD2, TDPSD5, TM, VAR, WL, ZC, mDWT	MEAN (10), AFB (9), mDWT (8), RMS (8), CEA (7), EndVal (7), ApEn (6), LOG (6), OHM (6), SNR (6), SSI (6), WAMP2 (6)
gyr	AFB, CEA, DFA, DMAV, DStd, DVAR, EndVal, KATZ, LOG, MAV, MAV2, MDF, MEAN, MYOP3, OHM, RMS, SNR, SSC, SSI, STD, StartVal, TP, V, WL, mDWT	MEAN (10), mDWT (10), SSI (9), CEA (8), DPR (8), DStd (8), MAV2 (8), AFB (7), DLD (7), EndVal (7), IEMG (7), LOG (7), MIN (7), RMS (7), MAV (6), MAX (6), SM (6), SNR (6), STD (6), V (6), WAMP2 (6)
emg	DMAV, FR, HG, SampEn, WAMP2	HG (10), WAMP2 (10), SampEn (7), ApEn (6)

Next to GM-Opt, multiple feature sets were constructed based on the occurrence of the features per modality. Ten feature sets were constructed (GM-01–GM-10) and compared with each other based on the performance on 80% of the MyPredict dataset. The average performance is shown in Figure 3B. It can be seen that, in terms of overall error, GM-05 performs best. Significant differences were found with GM-08, GM-09, and GM-10 (*p* < 1e−4, *p* < 1e−4, *p* < 1e−4, respectively). For transitional errors, GM-06 performs best. Significant differences were found with GM-01 and GM-10 (*p* = 0.044, *p* = 0.048, respectively). As no significant difference was found between GM-05 and GM-06 in terms of transitional and overall errors, we considered GM-06 to be the best feature set as this set contained less features than GM-05. GM-06 was used in the final evaluation.

### 3.3 State-of-the-Art Features

**FIGURE 4:**
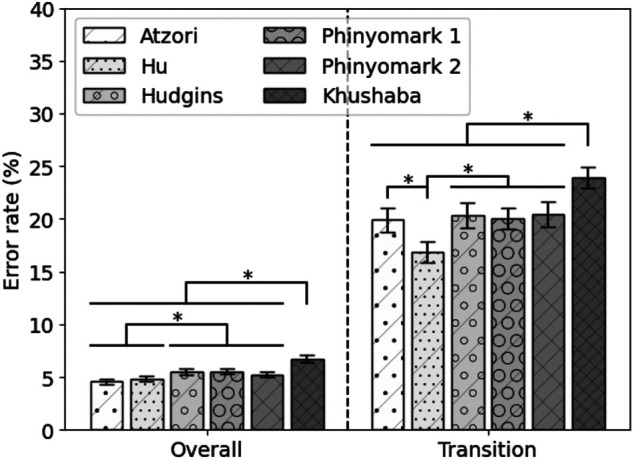
Average error rate (± SEM) of the state-of-the-art feature sets on 80% of the MyPredict dataset. A star indicates a significant difference.

The performance of the state-of-the-art feature sets on 80% of the MyPredict data is shown in Figure 4. The Atzori and Hu feature sets significantly outperform the Hudgins feature set (*p* = 1 < 1e−4, *p* = 0.002, respectively), Phinyomark 1 feature set (p < 1e−4, *p* = 0.001, respectively), Phinyomark 2 feature set (*p* = 0.001, *p* = 0.030, respectively), and Khushaba feature set (*p* < 1e−4, *p* < 1e−4, respectively). In terms of transitional error, the Hu feature set performs best, significantly outperforming the Atzori (*p* < 1e−4), Hudgins (*p* < 1e−4), Phinyomark 1 (*p* < 1e−4), Phinyomark 2 (*p* < 1e−4), and Khushaba (*p* < 1e−4) feature sets. The Khushaba feature set is significantly outperformed by the other feature sets (*p* < 1e−4). As there was no significant difference in terms of the overall error rate between the Atzori and Hu feature sets, but a significant difference in terms of the transition error rate, the Hu feature set was considered the best performing state-of-the-art feature set. The average error rates of the Hu feature set were 4.90 ± 0.25% and 16.89 ± 1.09% for overall and transitional errors, respectively, on 80% of the MyPredict dataset.

### 3.4 Final Evaluation

**FIGURE 5:**
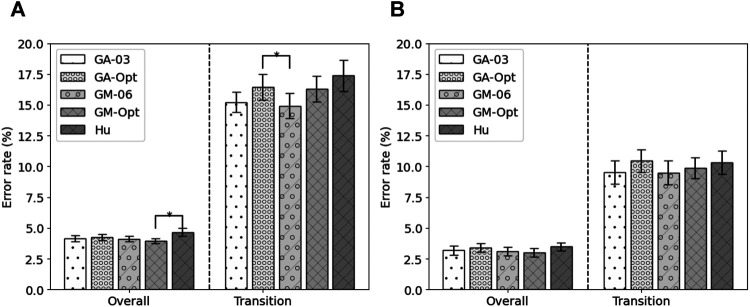
Average error rate (± SEM) of the selected feature sets on 20% of the MyPredict dataset **(A)** and on the ENABL3S dataset **(B)**. A star indicates a significant difference.

Five feature sets were selected for the final evaluation on the test sets: the remaining test set of the MyPredict dataset and the ENABL3S dataset. The five feature sets were the best performing state-of-the-art feature set (Hu), the feature set based on the best run of the genetic algorithm for all modalities (GA-Opt) and for each modality (GM-Opt), and the feature set constructed from the occurrence of selected features by the genetic algorithm for all modalities (GA-03) and per modality (GM-06). The error rates of these features on the test set of the MyPredict dataset are shown in Figure 5A, and the error rates on the ENABL3S dataset are shown in Figure 5B. Looking at Figure 5A, it can be seen that the GM-Opt feature set outperforms the Hu feature set (*p* = 0.048) in terms of overall error. The average overall error rates were 4.14 ± 0.23%, 4.24 ± 0.24%, 4.12 ± 0.22%, 3.94 ± 0.21%, and 4.66 ± 0.31% for GA-03, GA-Opt, GM-06, GM-Opt, and Hu, respectively. In terms of transitional error, GM-06 outperforms GA-Opt (*p* = 0.018). The average transitional error rates were 15.22 ± 0.82, 16.44 ± 1.03%, 14.92 ± 1.00%, 16.31 ± 1.04%, and 17.36 ± 1.28 for GA-03, GA-Opt, GM-06, GM-Opt, and Hu, respectively.

Looking at the error rates on the ENABL3S dataset in Figure 5B, it can be seen that the GM-Opt feature set outperforms the Hu feature set (*p* = 0.046) in terms of overall error. The average overall error rates were 3.18 ± 1.16, 3.40 ± 1.11, 3.10 ± 1.12%, 3.00 ± 1.05%, and 3.48 ± 1.07 for GA-03, GA-Opt, GM-06, GM-Opt, and Hu, respectively. The average error rates were 9.53 ± 2.98%, 10.45 ± 2.97%, 9.47 ± 3.08%, 9.88 ± 3.08%, and 10.32 ± 2.97% for GA-03, GA-Opt, GM-06, GM-Opt, and Hu, respectively.

## 4 Discussion

In this work, we investigated the use of genetic algorithms to construct optimized feature sets to be used in lower limb prosthetic control. After ten runs of the genetic algorithm on all modalities, two different optimized feature sets were constructed, GA-Opt and GA-03. Next to that, the genetic algorithm ran for 10 runs on each modality, and again, two feature sets were constructed, GM-Opt and GM-06. These four optimized feature sets were compared against the best performing state-of-the-art feature set, the Hu feature set, on two different datasets. All optimized feature sets outperformed the state-of-the-art feature set on the MyPredict dataset, and most optimized feature sets (except GA-Opt) outperformed state-of-the-art on the ENABL3S dataset. We considered GM-06 as the best performing feature set with the lowest transitional errors. GM-06 reduced the overall error from 4.66 to 4.12% and transitional error from 17.36 to 14.92% on the MyPredict dataset and reduced the overall error from 3.48 to 3.10% and transitional error from 10.32 to 9.47% on the ENABL3S dataset. GM-06 relatively decreased the overall error by 10.9–11.6% and transitional error by 8.2–14.1% compared with state-of-the-art on both datasets. Therefore, this study shows that feature selection using a genetic algorithm reduces overall and transitional errors, and it is worthwhile to use a genetic algorithm for feature selection in lower limb myoelectric control.

The results of this study show that even though the feature search space was greatly increased, the combined results of the genetic algorithm still outperformed the state-of-the-art feature sets. Other than the work by Phinyomark et al. (2017) or Qin and Shi (2020), we decided not to investigate the individual impact of each feature, as features combined may show added benefit in terms of performance. In our case, we wanted to have the genetic algorithm come up with a feature set, without looking into the groups features could belong to, to not restrict the genetic algorithm in its search. Compared to other studies involving meta-heuristics, we implemented over sixty different features which greatly increased the search space. For example, Luo et al. (2017) used ten features as input to their genetic algorithm, and Xi et al. (2018) used five features, which indicates a smaller search space than that used in this study. It can be concluded that the genetic algorithm is suited for feature selection within a vast search space.

The overall error rate of GM-06, 3.10–4.12%, is comparable with the results found in the literature. Spanias et al. (2018) reached an overall error rate of 4.03% in eight transfemoral amputees using a similar classification strategy to that in our work using the Hu feature set. Liu et al. (2017) reached an overall error rate of 4.2% in three able-bodied subjects and two transfemoral amputees, using mechanical information only. Su et al. (2019) used a convolutional neural network for classification and reached an overall error rate of 5.85% in ten able-bodied subjects. Halim et al. (2020) reached an average overall error of 6.0% using a random forest classifier on the ENABL3S dataset. Hu et al. (2018b) reported an average error rate of 2.09 ± 0.27% and 5.94 ± 0.84 for overall and transitional errors, respectively, using an ipsilateral sensor setup. These error rates are lower than the reported performance of the Hu feature set on the ENABL3S dataset in this study, 3.48 ± 1.07% and 10.32 ± 2.97% for overall and transitional error rates, respectively, although the same dataset was used for evaluation. The authors used a random 10-fold cross validation, which could decrease the error rate. This decrease is caused by steps that are close to each other in time and could end up in the separate training and test sets. This could cause the training and test sets to be more alike, which could in turn result in a lower error rate. We decided not to shuffle our data, to keep these steps close together, but this could have led to a slightly higher error than that reported by Hu et al. (2018b).

Phinyomark et al. (2017) clustered features based on their properties: features representing the amplitude and power of a signal (e.g., RMS), non-linear complex features (e.g., sample entropy), features representing frequency information (e.g., zero crossings, median frequency), time-series modeling (e.g., autoregressive coefficients), or unique features that capture a combination of information types (e.g., time-domain power spectral descriptors). The best performing optimized feature set based on all modalities was GA-03. It can be seen that most features for angular data are related to amplitude or power, except for mDWT (frequency), VCF (unique feature), and SampEn (non-linear complex). Acceleration features and angular velocity features contain more different feature types, although most of the selected features are related to amplitude and power. Looking at the kinematic features in the Hu feature set, we can see that all features fall in the amplitude and power groups. This could indicate that amplitude- and power-related features could be enough to represent kinematic data. When looking at GM-06, where features were selected per modality, we see something slightly different. Angle and angular velocity features are mostly amplitude or power related as seen before. Half of the features selected for acceleration are related to amplitude or power; however, frequency (mDWT), non-linear complex (ApEn, WAMP2), time-series modeling (SNR), and unique features (CEA, OHM) are seen as well. This suggests that acceleration feature sets could be improved by using features from different feature groups as well.

The number of EMG features is limited in GA-03, but mostly features related to the non-linear complex group are seen. Two out of five features coming from the non-linear complex group (WAMP2, KATZ) and one other feature are also related to entropy (PSDFD). The EMG features in GM-06 are all from the non-linear complex group, and most are based on entropy (HG, SampEn, and ApEn). This corresponds to the findings reported by Phinyomark et al. (2017), as they indicated that SampEn would be the most useful feature for EMG if used by itself. These findings support the idea that EMG is a non-linear signal and could best be represented by non-linear features, such as entropy-related features.

When looking at Table 3, it can be seen that relatively many angular features were used, whereas the number of EMG features was limited. This suggests that the data from angular features and to a lesser extent the acceleration and angular velocity features contain more useful information than EMG features. The most selected angular feature was EndVal, which selected eight out of ten runs. For EMG, it was WAMP2, which selected only five out of ten times. One explanation is that EMG features would be interchangeable due to the stochastic nature of EMG, and thus, no EMG features had as high occurrence as angular features. When looking at Table 4 in combination with the fitness values per modality, we can see something similar. Modality angle and acceleration had the highest fitness values of 0.738 and 0.731, respectively, indicating the highest performance. Joint angle information could be captured with a limited amount of features while reaching a high performance, whereas more features were necessary for acceleration data. The angular velocity needed many features as well, for a slightly worse performance. EMG could be captured with a limited number of features. However, EMG performed much worse than the other three modalities. This indicates that EMG by itself will not result in an optimal performance but still has some added benefit as seen in the literature as well. Young et al. (2014) showed that adding EMG decreased the transitional error from 18 to 12%, although EMG by itself would result in transitional errors of around 25%. Tkach and Hargrove (2013) showed that adding EMG to mechanical sensor data reduces the overall mean error from 7.8 to 2.3%, but EMG by itself results in overall errors of more than 30%. Spanias et al. (2015) showed that adding EMG did reduce the transitional error by approximately 3%, but adding additional mechanical sensor information reduced the transitional error further by almost 6%, resulting in a transitional error of approximately 13%. However, in clinical settings, the added effect of EMG can be doubted according to Liu et al. (2017), due to the large variability of EMG and susceptibility to movement and varying conditions. Therefore, they decided not to measure EMG at all and still reached low error rates of around 4.2% in an amputee population. In another work, Spanias et al. (2018) showed that the transitional error decreased by adding EMG to the sensor setup when using the non-adaptive version of their algorithm. However, no significant differences were seen in the error rate between mechanical sensors only and the addition of EMG when using their adaptive algorithm, although they reached lower error rates compared with the non-adaptive algorithm. This would indicate that a better classifier strategy has more influence on the error rate than adding EMG information. If we relate the findings in the literature to those in our work, this would indicate that the reason for an under-representation of EMG features is mostly due to the limited information one could get from EMG.

Compared with other feature sets in the literature, the found feature set is quite large and contains features with a high computational load, such as entropy-based features. This might impact the computational time and the computational load of a prosthesis, possibly increasing the energy requirement and introducing a delay. However, as microprocessors get more energy efficient and more computationally powerful, we do not expect the computational load to be a bottleneck. Next to this, the energy requirement for torque generation is an order of magnitude larger, and one could expect that a microprocessor processing a large feature set does not have a large impact on the energy consumption of a prosthetic device overall.

We decided to implement a genetic algorithm with one addition, heterogeneity. Heterogeneity was implemented to ensure that the population would not converge too quickly to one solution. The downside of this approach is that the room for potential better solutions could be limited by introducing random solutions. However, when looking at Figure 2, it can be seen that solutions quickly converge to the maximum fitness, and thus, the difference between the median and maximum fitness is reduced. By introducing random samples to the population, the population became more diverse for a couple of generations and leading to better solutions. After a couple of generations, the population seems to converge again and the process starts over. This resulted in the “spikes” which are visible in the image. Would we not have implemented this strategy, the population would quickly converge to a solution that would be possibly less optimal, and by introducing random samples, the genetic algorithm is able to search outside this local optimum.

In this study, we have focused on the genetic algorithm, although many more meta-heuristics exist. It is important to identify potential meta-heuristics that are suitable for feature selection. A broad range of metaphor-based meta-heuristics can be seen in the field, ranging from spotted hyena optimizers, elephant herding optimizers, to super-bug algorithms and raindrop algorithms. These “novel” optimizers are not novel at all, but just adaptations of already existing meta-heuristics (Sorensen et al., 2017). Hence, the decision to stick with a known and often used meta-heuristic.

A limitation of our study compared with the aforementioned studies is that our evaluation is performed on able-bodied subjects, although application is aimed toward a population with a gait impairment, such as amputees. Therefore, the question arises whether these results can be translated toward a population with gait impairments. Next to their evaluation on able-bodied subjects, Hu et al. (2018b) implemented their classification strategy in a prosthesis as well and showed that similar performance can be reached in an amputee population. Therefore, it can be expected that the classification strategy would work equally well in a population with impairments. Another limitation of our study is that we did not investigate the influence of sensor selection on the error rate. One could expect based on the literature that, by using sensor selection, the error rate could be reduced even further, such as shown by Young et al. (2014), or that the computational time can be reduced while not decreasing the error rate as shown by Halim et al. (2020). Sensor selection next to feature selection could be performed by a genetic algorithm, but this would greatly increase the search space, and thus, the question would arise again whether this search space is not too large. However, results from our study encourage finding the limitations of a genetic algorithm or other meta-heuristics in terms of feature and sensor selection.

## 5 Conclusion

The goal of this study was to investigate the use of a genetic algorithm for feature selection to enhance myoelectric control of the lower limb. The optimized feature set GM-Opt and the constructed feature set GA-03 and GM-06 both reduced error rates compared with state-of-the-art feature sets on two different databases. GM-06 outperformed the state-of-the-art feature set by relatively reducing overall errors up to 11.6% and transitional errors up to 14.1%, although these results were not significant. This study shows the importance and potential of feature selection in myoelectric control of the lower limb and the suitability of a genetic algorithm to search a large feature space.

## Data Availability

The raw data supporting the conclusions of this article will be made available by the authors, without undue reservation.
